# Effects of Antioxidants in Human Milk on Bronchopulmonary Dysplasia Prevention and Treatment: A Review

**DOI:** 10.3389/fnut.2022.924036

**Published:** 2022-07-18

**Authors:** Xianpeng Yang, Shanyu Jiang, Xianhui Deng, Zichen Luo, Ailing Chen, Renqiang Yu

**Affiliations:** ^1^Department of Neonatology, Wuxi Maternity and Child Health Care Hospital, Wuxi School of Medicine, Jiangnan University, Wuxi, China; ^2^Translational Medicine Laboratory, Research Institute for Reproductive Health and Genetic Diseases, The Affiliated Wuxi Maternity and Child Health Care Hospital of Nanjing Medical University, Wuxi, China

**Keywords:** bronchopulmonary dysplasia, premature infants, oxidative stress, human milk, antioxidants

## Abstract

Bronchopulmonary dysplasia (BPD) is a severe chronic lung illness that affects neonates, particularly premature infants. It has far-reaching consequences for infant health and their families due to intractable short- and long-term repercussions. Premature infant survival and long-term quality of life are severely harmed by BPD, which is characterized by alveolarization arrest and hypoplasia of pulmonary microvascular cells. BPD can be caused by various factors, with oxidative stress (OS) being the most common. Premature infants frequently require breathing support, which results in a hyperoxic environment in the developing lung and obstructs lung growth. OS can damage the lungs of infants by inducing cell death, inhibiting alveolarization, inducing inflammation, and impairing pulmonary angiogenesis. Therefore, antioxidant therapy for BPD relieves OS and lung injury in preterm newborns. Many antioxidants have been found in human milk, including superoxide dismutase, glutathione peroxidase, glutathione, vitamins, melatonin, short-chain fatty acids, and phytochemicals. Human milk oligosaccharides, milk fat globule membrane, and lactoferrin, all unique to human milk, also have antioxidant properties. Hence, human milk may help prevent OS injury and improve BPD prognosis in premature infants. In this review, we explored the role of OS in the pathophysiology of BPD and related signaling pathways. Furthermore, we examined antioxidants in human milk and how they could play a role in BPD to understand whether human milk could prevent and treat BPD.

## Introduction

BPD is the most prevalent complication in preterm infants. In 1967, Northway et al. first defined BPD as a persistent lung injury caused by a high concentration of oxygen and high pressures during mechanical ventilation. “Classical BPD” or “old BPD” is diagnosed in “those newborns who require supplementary oxygen on post-natal day 28” and those who display radiographic abnormalities in the chest, such as emphysema, atelectasis, and vesical shadows (1). The survival rate of premature infants has improved dramatically in the decades since BPD was identified, owing to a substantial increase in the quality of nursing for premature infants, such as the development of non-invasive ventilation techniques and prenatal use of corticosteroids for increased alveolar surface area. However, the incidence of BPD remains relatively high. Moreover, the advancement of these tools has increased our understanding of BPD; the definition of “old BPD” is no longer appropriate for current diagnostic criteria.

In 2001, Jobe et al. updated the diagnostic criteria and definition of BPD (NIH), also known as “New BPD”: infants with oxygen dependence (inhaled oxygen concentration [FiO_2_] >21%) for >28 days should be diagnosed with BPD, and classified as mild, moderate, severe BPD based on aerobic conditions at 36 week post-menstrual age (PMA). The new BPD highlights the link between alveolar dysplasia and lung injury in preterm infants (2, 3). In 2018, the National Institute of Child Health and Human Development modified its rules, removing the need for oxygen dependence for >28 days (4). In 2019, Jensen et al. proposed that the degree of oxygen reliance should not be considered when diagnosing BPD; instead, the mode of assisted breathing should be considered (5). For clinicians, an accurate diagnosis of BPD would allow the development of individualized respiratory support measures and medication therapy, which are advantageous for the rehabilitation and prognosis of patients with BPD.

Between 2010 and 2019, the incidence of BPD among extremely premature newborns in China was >74%; the younger the PMA, the higher the incidence of BPD (6). However, the treatment of BPD is controversial. A worse prognosis usually accompanies a higher disease severity. Currently, the most common treatment options for BPD are delivery room intervention (7, 8), invasive mechanical ventilation (9, 10), steroids (11), diuretics (12), bronchodilators (13), and caffeine (14, 15). Furthermore, dietary support, infection management, and vasodilators are beneficial as therapy for BPD (16). The importance of OS in the etiology of BPD has been increasingly recognized, and the use of antioxidants in the treatment of BPD is becoming a popular research topic. Reducing the damage caused by oxidative free radicals to the lungs of preterm infants by maintaining the balance between oxidation and antioxidant systems is expected to provide a new method for preventing and treating BPD (17, 18).

Human milk is the most comprehensive and natural nourishment for infant growth. It contains all the nutrients and biologically active components that newborns require to meet their developmental demands and boost their immunity. Breast milk contains various antioxidants, such as glutathione (GSH), superoxide dismutase (SOD), glutathione peroxidase (GSH-Px), vitamins, phytochemicals, melatonin, probiotics, short-chain fatty acids (SCFAs), and unique human milk oligosaccharides(HMOs), milk fat globule membrane(MFGM), and lactoferrin, which provide a solid antioxidant capacity for newborns. A meta-analysis on breastfeeding and BPD found that the incidence of BPD decreased when premature newborns were exclusively breastfed compared to when they were formula fed (19). In a cohort study, the amount of human milk consumed by premature newborns was inversely associated with the incidence of BPD, with a daily intake of 7 ml/(kg·d) showing a BPD prevention effect (20). Aloka et al. found that providing sufficient nutritional care to premature children with low birth weight is significant for preventing BPD. For every 10% increase in breastfeeding in preterm children from birth to 36 weeks PMA, the risk of BPD is lowered by 9.5% (21). Breastfeeding also reduces the risk of newborn problems in the neonatal intensive care unit (NICU) (22). Yan et al. discovered that a daily intake of 50 ml/(kg·d) of human milk from the mother during the first 4 weeks after delivery can minimize the incidence of BPD in infants (23). According to these studies, human milk appears to serve a beneficial function in the prevention and treatment of BPD. Given the presence of different antioxidants in breast milk, we believe that this is the primary mechanism through which human milk contributes to decreased BPD. Therefore, this review examined the critical role of OS in BPD and the impact of antioxidants in human milk on the prevention and treatment of BPD. We have also discussed future considerations for clinical BPD treatment.

### Overview

**Figure 1:**
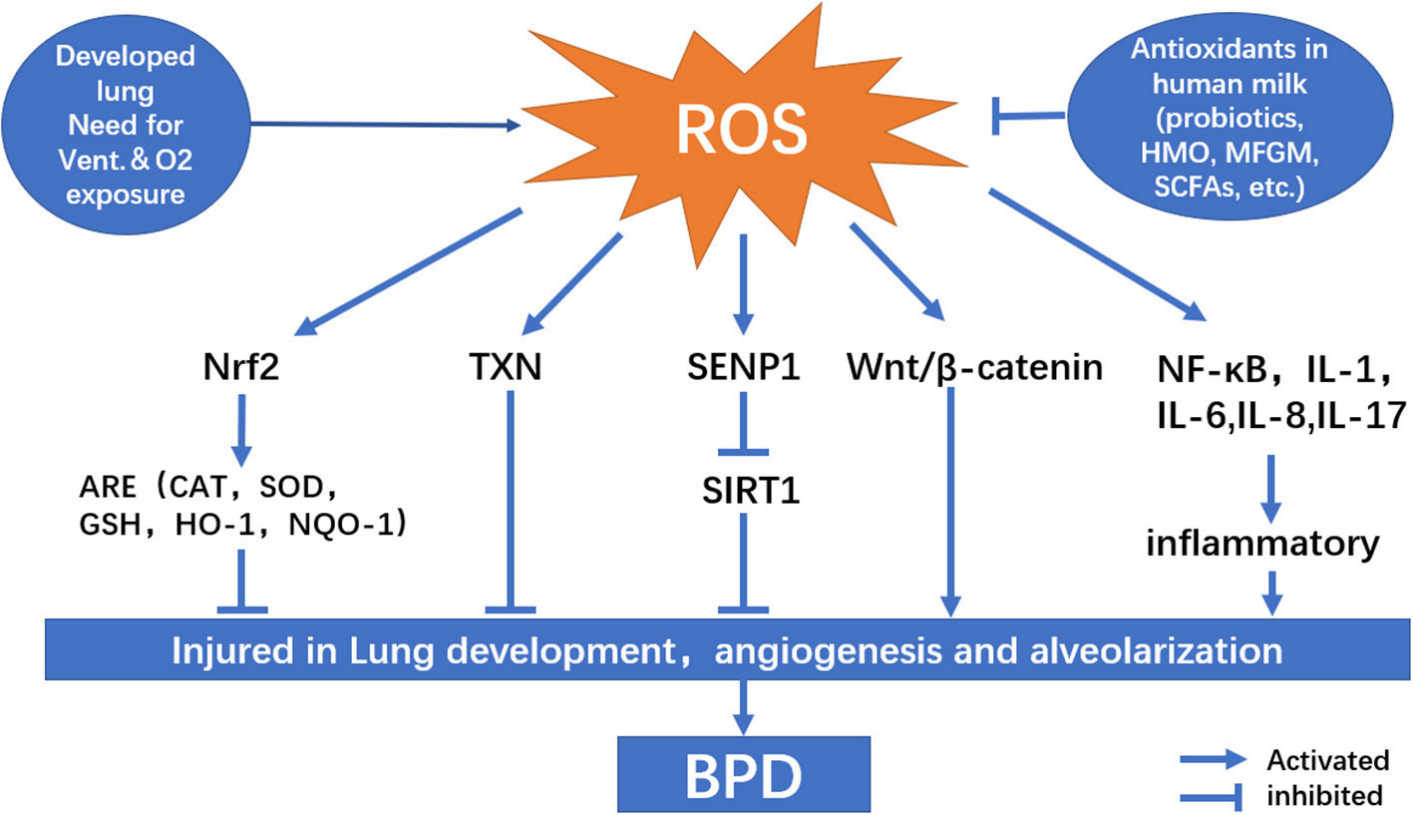
Signaling pathway related to oxidative stress on bronchopulmonary dysplasia pathogenesis.

OS is a condition in which there is an imbalance between reactive oxygen species (ROS) production and antioxidant capability. Under normal conditions, electrons created by the aerobic metabolism of cells are readily accepted by oxygen, resulting in ROS production. Excessive levels of ROS can harm cell structure, proteins, lipids, and nucleic acids, leading to cell death (24). The factors that influence the results of cell injury include interaction between specific molecules, the body's internal environment, cell location, time, and concentration (25). Premature newborns are more likely to develop BPD, especially those who require mechanical ventilation (Figure 1). Owing to respiratory system immaturity and pulmonary hypoplasia, premature infants require long-term oxygen therapy, which can expose them to a greater risk of illness and inflammation than term infants. They are also more likely to accumulate ROS due to high concentration oxygen dependence. Additionally, the endogenous antioxidant enzyme systems are defective in premature infants. SOD, catalase (CAT), and GPX concentrations at birth are substantially lower in premature newborns than in full-term infants; therefore, they are unable to effectively eliminate excess ROS, resulting in ROS accumulation and OS (26).

### OS and Lung Injury

#### Lung Development Injury

OS can cause several lung injuries. Long-term hyperoxia in the lungs of premature newborns causes lung damage directly through ROS production (27). Alveolar epithelium type II cells (AEC II) are the most critical cells in the lung development stage and act as lung tissue stem cells. AEC II synthesizes and secretes pulmonary surfactant, regulates lung tissue growth, repairs wounds, and adjusts alveolar moisture to maintain homeostasis. AEC II can develop into alveolar epithelial type I cells to repair the alveolar wall and preserve alveolar function when the alveolar–capillary barrier is damaged. A high concentration of oxygen can induce the apoptosis of AEC II and inhibit their proliferation. Injury to airway epithelial cells causes pulmonary tracheal remodeling and vascular remodeling, both of which are important in BPD etiology (28, 29). Airway remodeling occurs throughout infant lung development *via* several pathogenic processes involving airway epithelial cells, inflammatory cells, smooth muscle cells, and extracellular matrix. The leading causes of airway remodeling are aberrant airway healing and airway epithelial damage caused by decreased AEC II levels (30).

In addition, endoplasmic reticulum stress (ES) in the lungs can be exacerbated by OS in hyperoxic environments (31). Hyperoxia exposure can harm mitochondrial activity in the lungs, resulting in ES and alveolar injury, activating the pulmonary unfolded protein response (UPR), and increasing UPR downstream effectors, in turn resulting in alveolar growth disorder (32).

#### Arrested Alveolarization

Alveolarization (production of alveolar gas exchange units) can be impeded in newborns with BPD, although the mechanisms of alveolarization are unknown (33). Pulmonary alveoli are important gas exchange units, and alveolarization is a late goal in lung development. The terminal airspaces formed during early lung development are divided by secondary septation, resulting in an increasing number of smaller alveoli, thus improving gas exchangeability in the lungs. However, premature newborns with developed lungs are exposed to hyperoxia-obstructed gas exchange with continuous alternation of the bronchial, resulting in lung function disorder. In a previous study, neonatal mice treated with high oxygen (85% O_2_) showed delayed alveolar growth, loss of bronchoalveolar attachment, increased lung compliance, and increased medial arteriole wall thickness, which persisted into adulthood. Alveolar defects have also been linked to reduced functional gas exchange (34).

Supplemental oxygen levels influence the severity of alterations in airway anatomy and function in neonates. Mild (40% O_2_) and moderate (60% O_2_) oxygen exposure can cause alterations in airway function and hyperresponsiveness in animals, but the effects are minor. The degree of OS is directly associated with interrupted alveolarization due to severely high oxygen (80% O_2_) levels, which cause simplified alveolar, airway embolization, and elastin redistribution (35). Even a short period of hyperoxia lung injury (limited to cystic lung development) is sufficient to damage alveolar growth and reduce gas exchange function; irrespective of whether confined to the cystic period or chronic fetal hyperoxia, it will lead to reduced alveolar surface area volume and alveolar interval (36). Hyperoxia obstructs alveolarization, which is associated with mitochondrial malfunction. Both hyperoxia and direct suppression of mitochondrial oxidative phosphorylation can halt alveolar development. Long-term mechanical breathing inhibits the formation of pulmonary mitochondria, and this inhibition of oxidative phosphorylation persists throughout alveolar evolution, resulting in neonatal alveolar development delays (37, 38).

#### Pulmonary Vascular Injury

OS also impairs pulmonary angiogenesis. Pulmonary vascular abnormalities are another critical factor in BPD development; several growth factors play a role in pulmonary angiogenesis. The vascular endothelial growth factor (VEGF) can promote vascular endothelial cell proliferation and migration and reshaping of endothelial cells (39). VEGF also plays a crucial role in lung development by maintaining the typical structure and function of the alveoli during normal blood vessel development (40). OS caused by ROS accumulation has been confirmed to decrease VEGF expression in mouse models, and abnormal VEGF signal transduction can impair angiogenesis and reduce alveolarization, resulting in the arrest of pulmonary vascular development and leading to experimental BPD (41). Studies have shown that both VEGF expression and lung capillary density are significantly decreased in patients with BPD and animal models and that increased VEGF expression can inhibit alveolar destruction induced by high oxygen levels (42). The combination of mesenchymal stem cells (MSCs) and erythropoietin has been shown to enhance lung protection in newborn mice under high-oxygen conditions. Increased expression of VEGF was found to alleviate lung injury in newborn BPD mice by indirectly promoting angiogenesis (43). Wallace et al., using anti-sF1t-monoclonal antibody therapy found that sF1t, an endogenous antagonist of VEGF, could prevent structural abnormalities in lung development and pulmonary hypertension in infancy and effectively preserve lung structure and function (44). Thus, early endothelial cell injury damages pulmonary blood vessels and alveolar formation. In addition, downregulation of VEGF expression further disrupts lung epithelial and mesenchymal development. Premature infants, born with developed lungs and long-term exposure to high oxygen demand, develop ROS accumulation, resulting in OS and reduced VEGF expression. Under hyperoxia, reduced levels of VEGF cannot effectively induce pulmonary angiogenesis and repair, and pulmonary angiogenesis ceases during germination. Insufficient angiogenesis leads to pulmonary vascular dysplasia and prevents alveolarization, eventually leading to BPD (45).

### OS-Related Signaling Pathways

#### Nrf2–Keap1–ARE

Nuclear erythroid-E2-related factor 2 (Nrf2) is a major transcription factor involved in the antioxidant system that regulates OS. Nrf2 interacts with Keap1 in the cytoplasm and is destroyed by ubiquitination under normal conditions. The accumulation of ROS prevents Nrf2 from being ubiquitinated by modifying the cysteine in Keap1, allowing Nrf2 to be expressed stably in the nucleus and activating the antioxidant reaction elements (ARE), which include the expression of a series of antioxidant genes such as *GSH, GCL, HMOX*, and *GSL* (46). The oxidative equilibrium maintained by Nrf2 is especially crucial to the respiratory tract after long-term exposure to hyperoxia in the lungs of preterm newborns. Lack of Nrf2 can increase the sensitivity and severity of different respiratory conditions such as BPD, adult respiratory distress syndrome, chronic obstructive pulmonary disease, asthma, and lung cancer, whereas activation of Nrf2 protects against various respiratory diseases (47). The Nrf2-Keap1-ARE pathway is one of the most crucial protective mechanisms against BPD, probiotics, SCFAs, lactoferrin, arginine can activate this pathway and show their antioxidative effects. The pulmonary tissue structure of mice exposed to hyperoxia becomes disordered, the alveolar wall begins to weaken as the hyperoxia exposure period increases, and the concentration of antioxidative stress-related enzymes (CAT, SOD, and GSH-Px) decreases under hypoxia condition. The Nrf2 content is significantly higher in hyperoxic mice than in normal oxygen-treated mice. In contrast, the level of Keap1 is lower, indicating that the body can upregulate Nrf2 expression by inhibiting Keap1 expression in hyperoxic mice; subsequently, Nrf2 enters the nucleus to activate ARE and improve antioxidant capacity (48).

Targeting Keap1 knockdown to increase endogenous Nrf2 expression can be an approach for preventing low alveolarization in preterm infants. Keap1 knockdown has been shown to improve lung cell proliferation after birth in neonatal mouse models, accompanied by an increase in the expression level of the antioxidant gene (nuclear GSH). These findings indicate that Nrf2 plays an essential role in the development of neonatal lung tissue (49). The Nrf2 pathway has several effects on oxidation regulation, such as increasing heme oxygenase-1 (HO-1) expression to improve antioxidant and anti-inflammatory ability. Its protection against development of BPD is mainly dependent on the enzymatic products carbon monoxide, bilirubin, and iron, which prevent alveolar simplification, improve vascular remodeling, and avoid lipid peroxidation (50). In addition, Nrf2 can activate NAD (P)H:quinone oxidoreductase 1(NQO1) to mitigate lung damage by inhibiting cell apoptosis in hyperoxia (51).

#### SENP1–SIRT1

Silent mating-type information regulation 2 homolog 1(SIRT1) is a deacetylase activated by nicotinamide adenosine dinucleotide (NAD+). It regulates gene expression by removing acetyl groups from proteins involved in processes such as apoptosis, inflammation, aging, and OS. Lack of SIRT1 can lead to a significant increase in ROS levels and inflammatory response, increasing NAD+ can activate SIRT1, such as resveratrol and some probiotics in breast milk. Small ubiquitin-like modifier (SUMO)-specific protease 1 (SENP1) regulates SIRT1 distribution in OS by participating in substrate de-SUMO modification (small ubiquitination related modification). SIRT1 undergoes SUMOylation, an essential post-translational modification. After SUMOylation, SIRT1 deacetylation is strengthened, and SIRT1 in the nucleus can suppress apoptosis by deacetylating p53. Through de-SUMO modification of SIRT1, SENP1 can reduce SIRT1 deacetylation. SIRT1 protein expression is considerably decreased in neonates with BPD, and the interaction between SUMO1 and SUMO2/3 is significantly weakened. These findings imply that a reduction in SIRT1 SUMOylation may play a role in the development of BPD (52–54).

Recent studies have shown that SIRT1 is linked to the pathogenesis of BPD (52). In peripheral blood mononuclear cells (PBMCs) of neonates with BPD, elevated ROS levels and upregulated SENP1 expression were found. Moreover, SIRT1 showed substantial spatial variations, with decreased expression in the nucleus and increased expression in the cytoplasm. SENP1 silencing can alleviate hyperoxic injury in the hyperoxic alveolar epithelial cell injury model, suggesting that SENP1 can inhibit SIRT1 deacetylation activity by regulating SIRT1 expression and distribution in the nucleus, promoting cell apoptosis, and playing an essential role in hyperoxia-induced lung injury (55). In newborns with BPD, nuclear SIRT1 expression in PBMCs collected by tracheal aspiration is lower and nuclear SIRT1 localization is substantially lower than those in normal neonates (56). The level of ROS and pace of SIRT1 translocation in premature newborns receiving assisted oxygen are strongly oxygen concentration dependence and are associated with a reduction in SIRT1 expression, suggesting that OS can change the activity and distribution of SIRT1. Resveratrol inhibits ROS formation under hyperoxia exposure by blocking the SIRT1 nuclear plasma shuttle and increasing SIRT1 expression (57). Furthermore, through the SIRT1 pathway, budesonide and porcine lung phospholipid injection can exert a protective effect on premature BPD (58).

#### Wnt/β-Catenin

The Wnt/β-catenin signaling cascade is critical for lung formation during early stages. Wnt signaling occurs at 7 weeks of pregnancy, peaks at 17 weeks, and subsequently declines at 21 weeks (59). According to Zhang et al., Wnt transcription factors are found in low amounts in the respiratory epithelium and mesenchymal lining of the lungs. It plays a vital role in regulating lung development. In neonatal rat lung injury caused by hyperoxia, abnormal activation of Wnt/β-catenin and decreased expression of peroxisome proliferator-activated receptor γ were observed. These changes were linked to reduction in alveolar septal thickness, radial alveolar count, and alveolar augmentation after hyperoxia exposure (60). Alapati et al. found that inhibiting β-catenin expression increased alveolar remodeling and reduced the incidence of pulmonary vascular remodeling and pulmonary hypertension in newborn rats, suggesting the involvement of Wnt/β-catenin pathway in hyperoxia-induced alveolar damage (61).

The expression time and regulation of Wnt signaling during lung development are minimal, and the activation of an aberrant Wnt gene can hinder normal lung development. The development of BPD is complicated by exposure of the lung to high oxygen levels during the cystic stage (62). Jennifer et al. discovered that at the cystic stage, increased oxygen exposure causes aberrant activation of mesenchymal Wnt5A. Furthermore, a three-dimensional organotypic coculture system has revealed that Wnt/β-catenin signal transduction occurs in AEC II and fibroblasts and that targeted suppression of Wnt5A can alleviate hyperoxia-induced alveolar constriction. There are several approaches to ameliorate hyperoxia-induced alveolar epithelial cell injury (YAP and ETS1) *via* the Wnt/β-catenin signaling pathway. However, given that the Wnt/β-catenin signaling pathway is essential for normal lung development, targeted therapy for this pathway is controversial. To ensure lung development and injury repair in premature infants, it is essential to accurately match the treatment objectives to restore the balance of the lung development signaling pathway (63, 64).

#### TXN System

Thioredoxin (TXN) is a small-molecule protein that functions as a hydrogen carrier in cells. Thioredoxin reductase (TXNRD) is a dimer released with a NAPDH dependent FAD domain. The TXN system, which includes TXN, TXNRD, and NAPDH, regulates REDOX equilibrium, cell proliferation, and cell death. It is also involved in embryonic development and immunological metabolism (65). TXN and TXNRD are primarily expressed in pulmonary epithelial cells, alveolar macrophages, and bronchial chondrocytes in neonates, and they function as antioxidants (66). Recent studies have linked TXN system damage with hyperoxic injury and abnormal lung development, noting that the TXN system not only has a simple ROS detoxification function but also acts as a REDOX sensor when oxygen concentration changes, assisting newborns to complete the oxygen concentration conversion process from intrauterine relative hypoxia to hyperoxia exposure (67).

The TXN system efficiently prevents oxidative damage and contributes to REDOX-sensitive lung growth signals. TXN1 siRNA knockdown reduces cell viability after hyperoxia therapy, whereas TXN1 overexpression increases cell survival (68). To minimize lung injury in the BPD model, the antioxidant capacity of the TXN system is dependent on the activation of Nrf2 and an increase in lung epithelial HO-1 expression (69). According to Zhang et al., TXN1 overexpression reduced apoptosis and increased MSC proliferation in lung MSCs transplanted into recipients, SOD levels, and GPX levels (70). TXN1 is a new target for BPD treatment in neonatal lung disorders as it is resistant to hyperoxic lung injury. Increased expression levels of TXN1, TXN2, and TXNRD have been reported in hyperoxia. In GSH reductase-deficient mice, the TXN system showed an apparent antioxidant compensatory effect (71). Wall et al. found that using aurothioglucose (ATG) as a TXNRD1 inhibitor increased the total GSH content and GPX activity of ATG-treated mice under hyperoxia, suggesting that ATG could improve GSH-dependent antioxidant capacity (72). TXNRD inhibitors also have favorable effects on lung development. RNA sequencing in the lungs of mice exposed to >95% oxygen for the first 72 h of life revealed that ATG selectively controlled genes were primarily related to angiogenesis and vascular development and that ATG use increased pulmonary vascular density in mouse models (73).

#### NF-κB

OS stimulates nuclear factor κB (NF-κB), which regulates inflammatory factor synthesis and activation, nuclear protein translocation, and apoptosis (74). NF-κB is a significant regulator of the incidence, progression, and clearance of inflammation and plays a prominent role in lung inflammation. Although phosphorylation of IκBα (inhibitor of NF-κB α) and nuclear accumulation of NF-κB P65 have been linked to the severity of BPD in recent studies, the role of NF-κB in BPD is debated (75). In breast milk, melatonin, HMOs, and lactoferrin can regulate oxidative balance through NF-κB. OS simultaneously triggers the body's inflammatory response. When exposed to hyperoxia, monocytes, macrophages, lymphocytes, epithelial cells, endothelial cells, and the matrix produce many chemokines, resulting in an influx of inflammatory cells. These are the most critical factors in the mechanism of hyperoxia-induced lung injury. Late chronic inflammatory injury is an essential factor that leads to abnormal lung development (76). OS stimulates several pro-inflammatory factors and mediators, such as IL-1, IL-6, IL-8, IL-17, IL-24, MCP-1, and TNF-α, resulting in an inflammatory cascade (77–81). Inflammation levels in neonates with BPD continue to rise after birth, progressively diminishing after 2 weeks. This trend is particularly pronounced in males and infants with very low birth weight, implying that more targeted treatment interventions should be implemented early in BPD (82).

Previous research has shown that persistent hyperoxia-induced NF-κB activation helps survival and lung growth in neonates. NF-κB activation contributes to the activation of cell protection target genes such as VEGF receptor 2. Inhibition of NF-κB expression in the developing lung lowers vascular proliferation and alveolar–capillary density. It produces reduced alveolarization similar to BPD, indicating that NF-κB activation can be a therapeutic intervention to prevent BPD (83, 84). However, recent studies have suggested that inhibition of NF-κB can reduce hyperoxia-induced newborn lung injury. Tetralin (Tet) enhances antioxidant levels and lowers inflammatory factor levels in hyperoxia-induced lung function decline in rats. Jiao et al. have reported that Tet achieves this by suppressing NF-κB (85). Li et al. used a recombinant human elastase inhibitor, elafin, to inhibit the NF-κB pathway in hyperoxia-exposed mice. They found that elafin reduced apoptosis, inhibited inflammatory cytokines, improved nuclear accumulation of NF-κB P65, and improved alveolarization (86). According to Chen et al., caffeine can also reduce nuclear apoptosis and inflammatory lung injury in lung tissue, lower OS levels, enhance alveolar growth, and protect lung development from oxidative damage in hyperoxia (87). Excessive activation or inhibition of the NF-κB pathway is detrimental to the normal development of the lungs, and direct targeting of the NF-κB pathway may further lead to pulmonary dysplasia. New methods for the prevention and treatment of BPD include targeting NF-κB signaling and suppressing dangerous downstream signals of the NF-κB pathway.

Therefore, OS plays a significant role in the etiology of BPD. Antioxidant treatment can lower infant lung OS, using that antioxidant treatment is an essential study direction for BPD. However, as the free radicals produced by BPD are diverse, the clinical effect of a single antioxidant treatment for BPD does not achieve the desired effect. Therefore, applying various antioxidants in breast milk may positively affect the prevention and treatment of BPD.

## Antioxidants in Breast Milk

Human milk is the safest and most natural nourishment for infants (88). It contains all the calories, proteins, and lipids that newborns require for growth and development and is also high in antioxidants (Table 1). Antioxidants are thought to exist in a range of active compounds. They can remove ROS or reactive nitrogen species directly, change the activity of enzyme boosting and antioxidant enzymes, or regulate REDOX signaling pathways to achieve their antioxidant actions. The overall antioxidant capacity of breastmilk is an important defense mechanism for preventing illnesses that affect newborns (Table 2). Breastfeeding has been shown to minimize the incidence of BPD in premature newborns in clinical practice.

**Table 1:** Antioxidants in human milk and differences between term (PMA > 37 weeks) and preterm (PMA < 37 weeks) newborn mothers' milk.

**Substances and lactation stage**	**Value and unit in term infant HM**			**Value and unit in preterm infant HM**		**References**
**Total HMO**						
Colostrum^a^	Mean ± SD^d^	12.5 ± 7.2^d^	g/L	/		(89)
		9.6 ± 6.1		/		(90)
		22.4 ± 4.6		/		(91)
		13.0 ± 3.9		/		(92)
Transitional milk^b^	Mean ± SD	11.0 ± 6.0	g/L	/		(89)
		8.4 ± 5.0 18.9 ± 3.9		/ /		(90) (91)
		10.7 ± 2.1		/		(92)
Mature milk^c^	Mean ± SD	9.6 ± 5.1	g/L	/		(89)
		6.6 ± 4.4		/		(90)
		14.6 ± 4.3		/		(91)
		9.2 ± 2.0		/		(92)
**2** **′** **FL**						
Colostrum	Mean ± SD	3.57 ± 1.90	g/L	2.50 ± 1.63	g/L	(93)
Transitional milk	Mean ± SD	2.20 ± 1.43	g/L	1.67 ± 1.12	g/L	(93)
		1.00 ± 0.06		/		(94)
Mature milk	Mean ± SD	2.00 ± 1.25	g/L	1.65 ± 1.47	g/L	(93)
	Mean	2.74		2.77		(95)
	Mean ± SD	0.84 ± 0.57		/		(94)
**LNnT**						
Colostrum	Mean ± SD	0.33 ± 0.09	g/L	0.27 ± 0.12	g/L	(93)
Transitional milk	Mean ± SD	0.22 ± 1.00	g/L	0.22 ± 0.09	g/L	(93)
		1.22 ± 0.47		/		(94)
Mature milk	Mean ± SD	0.17 ± 0.08	g/L	0.18 ± 0.08	g/L	(93)
	Mean	0.74		0.66		(95)
	Mean ± SD	1.00 ± 0.42		/		(94)
**3** **′** **–GL**						
Colostrum	Mean ± SD	12.96 ± 6.37	mg/L	11.69 ± 8.00	mg/L	(93)
Transitional milk	Mean ± SD	7.13 ± 4.93	mg/L	6.21 ± 4.19	mg/L	(93)
Mature milk	Mean ± SD	5.54 ± 3.06	mg/L	5.58 ± 5.48	mg/L	(93)
**Vitamin A**						
Colostrum	Range	5–7	μmol/L	3	μmol/L	(96)
	Mean ± SD	146.9 ± 70.9	μg/100 mg	/	μmol/L	(97)
Transitional milk	Range	3–5	μmol/L	3.5	μmol/L	(96)
	Mean ± SD	81.8 ± 45.8	μg/100 mg	/		(97)
Mature milk	Range	1.4–2.6	μmol/L	2	μmol/L	(96)
	Mean ± SD	1.87 ± 0.81		1.38 ± 0.67		(98)
	Mean ± SD	1.76 ± 0.85		/		(99)
	Mean ± SD	59.5 ± 51.6	μg/100 mg	/		(97)
**α** **tocopherol**						
Colostrum	Mean ± SD	9.99 ± 1.51	mg/L	/	mg/L	(100)
		/		7.77 ± 2.88		(101)
	Mean ± SD	612.6 ± 412.3	μg/100 g	/		(97)
	Median (Q1,Q3)^e^	840.40 (671.18,1157.02)	μg/100 ml	/		(102)
Transitional milk	Mean ± SD	4.45 ± 0.95	mg/L	/	mg/L	(100)
		/		4.68 ± 2.94		(101)
	Mean ± SD	248.5 ± 218.5	μg/100 g	/		(97)
	Median (Q1,Q3)	418.68 (322.39,535.20)	μg/100 ml	/		(102)
	Mean ± SD	2.92 ± 0.84	mg/L	/		(100)
	Median (Q1,Q3)	3.16 (2.29,4.16)		/		(103)
Mature milk	Mean ± SD	177.1 ± 109.0	μg/100 g	/		(97)
	Median (Q1,Q3)	290.6 (223.98,382.00)	μg/100 ml	/		(102)
**γ** **tocopherol**						
Colostrum	Mean ± SD	0.57 ± 0.21	mg/L	/		(100)
	Median (Q1,Q3)	110.07 (72.93,165.14)	μg/100 ml			(102)
Transitional milk	Mean ± SD	0.60 ± 0.21	mg/L	/		(100)
	Median (Q1,Q3)	76.78 (47.27,113.42)	μg/100 ml	/		(102)
Mature milk	Mean ± SD	0.30 ± 0.14	mg/L	/		(100)
	Median (Q1,Q3)	0.89 (0.58,1.27)		/		(103)
	Median (Q1,Q3)	57.28 (35.60,84.70)	μg/100 ml			(102)
**SCFA**						
Formate (C1:0)						
Mature milk	Mean (range)	43.7 (15.2–4,960.3)	μmol/L	/		(104)
Acetate (C2:0)						
Mature milk	Mean (range)	46.8 (13.5–4,307.7)	μmol/L	/		(104)
Butyrate (C4:0)						
Colostrum	Mean ± SD	0.06 ± 0.06	mg/g milk fat	0.12 ± 0.11	mg/g milk fat	(105)
Transitional milk	Mean ± SD	0.07 ± 0.05	mg/g milk fat	0.12 ± 0.09	mg/g milk fat	(105)
Mature milk	Mean ± SD	0.24 ± 0.20	mg/g milk fat	0.09 ± 0.07	mg/g milk fat	(105)
	Mean (range)	95.6 (4.8–409.5)	μmol/L	/		(104)
**Lactoferrin**						
Colostrum	Range	6–8	g/L	/	g/L	(106)
	Mean	3.16		/		(107)
	Mean ± SD	/		14.92 ± 7.96		(108)
	Mean	0.37		0.76		(109)
Transitional milk	Mean	1.73	g/L	10.73 ± 5.67	g/L	(107)
	Mean ± SD	/		0.58		(108)
	Mean	0.37				(109)
Mature milk	Range	1–2	g/L	/	g/L	(106)
	Mean	0.90		/		(107)
	Mean ± SD	/		10.34 ± 6.27		(108)
	Mean	0.30		0.39		(109)
	Mean ± SD	3.39 ± 1.43		/		(109)
**Melatonin**						
Colostrum (nighttime)	Mean	25.31	pg/ml	28.67	pg/ml	(110)
	Median (Q1,Q3)	36.9 (19.1,48.7)		/		(111)
Transitional milk (nighttime)	Mean	22.55	pg/ml	24.70	pg/ml	(110)
	Median (Q1,Q3)	30.8 (12.4,37.8)		/ /		(111)
	Median (Q1,Q3)	70.7 (26.3,129.7)				(112)
Mature milk (nighttime)	Mean	20.12	pg/ml	22.37	pg/ml	(110)
	Median (Q1,Q3)	32.6 (15.6–47.9)		/		(111)
	Median (Q1,Q3)	31.3 (23.2,51.1)		/		(112)
	Mean (range)	3.9 (0.8–36.2)		2.7 (0.1–30.5)		(113)
**Phytochemicals** **epicatechin**						
Transitional milk Mature milk	Mean (range)	90.5 (68.3–120.5) 249.2 (63.7–828.5)	nm/L	/ /		(114)
**Epicatechin gallate**						
Transitional milk Mature milk	Mean (range)	189.5 (55.7–609.3) 236.6 (62.2–645.6)	nm/L	/ /		(114)
**Epigallocatechin gallate**						
Transitional milk Mature milk	Mean (range)	1,118.8 (425.5–2,364.7) 667.2 (215.1–1,683.8)	nm/L	/ /		(114)
**Naringenin**						
Transitional milk Mature milk	Mean (range)	251.2 (82.9–542.6) 210.4 (98.1–722.0)	nm/L	/ /		(114)
**Kaempferol**						
Transitional milk Mature milk	Mean (range)	15.7 (7.8–34.0) 23.1 (8.9–53.6)	nm/L	/ /		(114)
**Hesperetin**						
Transitional milk Mature milk	Mean (range)	459.2 (107.1–1,272.8) 393.6 (79.9–1,603.1)	nm/L	/ /		(114)
**Quercetin**						
Transitional milk Mature milk	Mean (range)	48.1 (40.0–77.6) 59.8 (33.1–108.6)	nm/L	/ /		(114)
**α-carotene**						
Transitional milk Mature milk	Mean (range)	59.0 (12.0–220.6) 19.2 (7.3–46.5)	nm/L	/ /		(114)
**β-carotene**						
Colostrum	Median (Q1,Q3)	11.14 (6.47,23.27)	μg/100 ml	/		(102)
Transitional milk	Mean (range)	164.3 (17.3–327.8)	nm/L	/		(114)
	Median (Q1,Q3)	3.13 (1.78,5.3)	μg/100 ml	/		(102)
Mature milk	Mean (range)	104.4 (10.7–377.4)	nm/L	/		(114)
	Median (Q1,Q3)	1.77 (1.03,3.07)	μg/100 ml	/		(102)
**α-cryptoxanthin**						
Transitional milk	Mean (range)	30.6 (11.9–72.6)	nm/L	/		(114)
Mature milk	Mean (range)	16.8 (2.2–50.6)	nm/L	/		(114)
**β-cryptoxanthin**						
Colostrum	Median (Q1,Q3)	3.90 (1.54,7.02)	μg/100 ml	/		(102)
	Median (Q1,Q3)	754.6 (429.6,1,486)	nm/L	406.7 (231.4,853.0)	nm/L	(115)
Transitional milk	Mean (range)	57.4 (9.4–175.4)	nm/L	/		(114)
	Median (Q1,Q3)	1.99 (1.31,3.56)	μg/100 ml	/		(102)
Mature milk	Mean (range)	27.5 (2.1–70.7)	nm/L	/	nm/L	(114)
	Median (Q1,Q3)	190.6 (87.4,353.9)		135.1 (53.0,224.6)		(115)
	Median (Q1,Q3)	0.82 (0.46,2.08)	μg/100 ml	/		(102)
**Zeaxanthin**						
Colostrum	Median (Q1,Q3)	2.15 (1.27,3.34)	μg/100 ml	/		(102)
	Median (Q1,Q3)	106.4 (73.7,141.4)	nm/L	63.2 (35.9,112.6)	nm/L	(115)
Transitional milk	Mean (range)	46.3 (19.4–115.4)	nm/L	/		(114)
	Median (Q1,Q3)	2.21 (1.33,2.95)	μg/100 ml	/		(102)
Mature milk	Mean (range)	22.8 (11.9–52.6)	nm/L	/	nm/L	(114)
	Median (Q1,Q3)	59.9 (38.4,91.1)		46.6 (36.7,65.9)		(115)
	Median (Q1,Q3)	1.11 (0.7,1.93)	μg/100 ml	/		(102)
**Lutein**						
Colostrum	Median (Q1,Q3)	7.12 (5.13,13.03)	μg/100 ml	/		(102)
	Median (Q1,Q3)	486.3 (322.9,745.8)	nm/L	432.8 (231.6,667.9)		(115)
Transitional milk	Mean (range)	121.1 (58.1–412.9)	nm/L	/		(114)
	Median (Q1,Q3)	9.49 (6.77,13.1)	μg/100 ml			(102)
Mature milk	Mean (range)	61.9 (16.8–193.4)	nm/L	/	nm/L	(114)
	Median (Q1,Q3)	195.9 (150.3,270.8)		217.3 (170.0–283.0)		(115)
	Median (Q1,Q3)	4.57 (2.95,7.56)	μg/100 ml	/		(102)
**Lycopene**						
	Median (Q1,Q3)	11.88 (7.09,17.43)	μg/100ml	/		(102)
	Median (Q1,Q3)	1,065 (483.0,1846)	nm/L	669.9 (388.1,931.6)	nm/L	(115)
Transitional milk	Mean (range)	119.9 (30.5–317.5)	nm/L	/		(114)
	Median (Q1,Q3)	1.26 (0.96,2.52)	μg/100 ml	/		(102)
Mature milk	Mean (range)	58.0 (9.0–256.6)	nm/L	/	nm/L	(114)
	Median (Q1,Q3)	192.7 (118.8,221.2)		125.8 (89.4,173.6)		(115)
	Median (Q1,Q3)	0.58 (0.29,0.99)	μg/100 ml	/		(102)
**GSH**						
Transitional milk	Median (Q1,Q3)	20.2 (15.8,22.5)	Mg/ml	/		(112)
Mature milk		12.2 (10.5,15.8)		/		
**SOD**						
Colostrum	Mean ± SD	33 ± 15	units/ml	32 ± 20	units/ml	(116)
Transitional milk		43 ± 25		35 ± 16		
Mature milk		36 ± 11		37 ± 20		
Mature milk	Median (Q1,Q3)	202 (117–243)	ng/mol	245 (179–353)	ng/mol	(113)
**CAT**						
Transitional milk	Median (Q1,Q3)	261.5 (256.6,271.1)	units/ml	/		(112)
Mature milk		262.0 (253.5, 274.7)		/		
**GPX**						
Colostrum	Mean ± SD	9.0 ± 3.9	mU/mg	6.9 ± 1.6	mU/mg	(116)
Transitional milk		11.0 ± 4.3		10.0 ± 5.1		
Mature milk						
		10.1 ± 3.6		9.6 ± 2.5		
Mature milk	Median (Q1,Q3)	1,505 (799–2,269)	ng/mol	1,591 (1,174–2,208)	ng/mol	(113)
**Iron**						
Colostrum	Mean ± SD	0.11 ± 0.43	Mg/L	1.10 ± 0.34	Mg/L	(117)
Transitional milk		0.99 ± 0.31		0.99 ± 0.27		
Mature milk		0.88 ± 0.28		0.90 ± 0.23		
Mature milk	Mean (range)	313.0 (24.2–2,157.1)	μg/L	380.0 (110.5–3,594.0)	μg/L	(118)
**Copper**						
Colostrum	Mean ± SD	0.72 ± 0.13	Mg/L	0.83 ± 0.21	Mg/L	(117)
Transitional milk		0.73 ± 0.21		0.78 ± 0.18		
Mature milk		0.58 ± 0.09		0.63 ± 0.14		
Mature milk	Mean (range)	288.8 (81.6–829.0)	μg/L	618.3 (95.4–954.5)	μg/L	(118)
**Zinc**						
Colostrum	Mean ± SD	5.35 ± 1.20	Mg/L	5.30 ± 1.45	Mg/L	(117)
Transitional milk		4.10 ± 0.65		4.75 ± 1.56		
Mature milk		2.60 ± 0.65		3.92 ± 1.10		
Mature milk	Mean (range)	1,434.3 (76.3–9,632.0)	μg/L	2,614.4 (422.8–17,727.2)	μg/L	(118)
**Selenium**						
Mature milk	Mean (range)	8.4 (2.5–38.1)	μg/L	12.6 (3.0–70.6)	μg/L	(118)
**Arginine**						
Colostrum	Mean	94.3	μmol/L	/		(119)
Transitional milk		35.6		/		
Mature milk		30.2		/		
**Glutamine**						
Colostrum	Mean	13.5	μmol/L	/		(119)
Transitional milk		92.6		/		
Mature milk		134.6		/		

^*^Lactation Stage:

a *The earliest secreted milk after birth and in the first week postpartum (days 0–5)*.

b *The milk is expressed in the first or second week (days 6–15)*.

c *The milk secreted at the latest 4 weeks postpartum*.

d *Data are expressed as mean ± standard deviation (SD)*.

e *Data are expressed as median (interquartile range)*.

**Table 2:** Antioxidants in human milk and their antioxidative functions to BPD.

**Bioactive molecules**	**Antioxidative function**	**Clinical trials/formula**	**References**
**Probiotics**			
Lactobacillus Bifidobacterium	Metal chelation; Secretion of antioxidant enzymes and active antioxidant substances; Enhance antioxidant signaling pathway; Reduce free radical production; Protect gut microbiome	Supplement of Lactobacillus paracasei strain F19 in formula is safe and well-tolerated for newborns and reduces occurrence of upper respiratory infection (*n* = 200); Probiotics can reduce the incidence of BPD in preterm infants under 32 weeks gestational age (*n* = 318)	(120, 121)
**HMO**			
2′FL; LNnT; 3′-GL	Reduce free radical production; Protect gut microbiome	Infant formula supplemented with 2′FL (1 g/L) and LNnT (0.5 g/L) is safe, well-tolerated, and reduces the incidence of bronchitis (*n* = 88)	(122)
**MFGM**	Regulate intestinal flora	Adding bovine MFGM to formula is safe before newborn is 2 years old (*n* = 582); MFGM added to formula is safe and well-tolerated in neonates with few adverse reactions (*n* = 200)	(120, 123)
**SCFA**	Regulate intestinal flora; Anti-inflammation	/	
**Lactoferrin**	Inhibit lipid peroxidation; Enhance antioxidant signaling pathway	Enteral lactoferrin supplementation at 100 mg/day did not affect BPD morbidity and mortality in preterm infants with GA ≤ 32 weeks (*n* = 2,182).	(124)
**Melatonin**	Reduce free radical production; Promote production of antioxidant enzymes	Melatonin treatment reduce mortality and hospital stay of BPD in preterm infants (*n* = 80).	(125)
**Vitamin**			
Vitamin A Vitamin E	Reduce free radical production; Inhibit lipid peroxidation Reduce free radical production	Early oral administration of vitamin A (5,000 IU vitamin A/kg/ day) at 28 days postpartum in very low birth-weight infants reduced BPD morbidity and mortality (*n* = 457)	(126)
**GSH**	Reduce free radical production; Participate in Vitamin C and Vitamin E cycles	/	
**Phytochemicals** polyphenols carotenoids	Metal chelation; Reduce free radical production; Protect gut microbiome; Inhibition of oxidase; Converse to Vitamin A	/	
**SOD** **CAT** **GPX**	Catalyze transformation of superoxide anion free radicals into hydrogen peroxide Decomposition of hydrogen peroxide; Prevent accumulation of oxidative free radicals Decomposition of hydrogen peroxide; Avoid accumulation of oxidative free radicals	/	
**Free amino acid**	Synthesis NO; Promote GSH generation; Promote production of antioxidant enzymes	/	
**Trace element**	Catalyze REDOX reaction; Promote the antioxidant system	/	

### Probiotics

Probiotics are microbes that are beneficial to human health. Human milk contains a complex microbial community, providing probiotics and vital energy for neonates. The International Scientific Association for Probiotics and Prebiotics defined probiotic calibration in 2013 as “providing a health benefit to the host when administered in sufficient numbers of living microorganisms,” which includes microorganisms that have been shown to have a health benefit in controlled trials and new symbiotic strains from human samples (127). *Lactobacillus* and *Bifidobacterium* are the most prevalent probiotics found in human milk, which can dwell in the intestinal system of neonates along with other probiotics (128, 129). Chanettee et al. identified two *Lactobacillus* species, *L. plantarum* and *L. pentosus*, in human milk (130). Breast milk is the first source of intestinal bifidobacteria in newborns, and they dominate the intestinal flora of breastfed infants (131). The antioxidant capacity of probiotics can be observed in various ways. Probiotics can aid in the chelation of metal ions and removal of reactive free radicals. Their metal chelation action is strain specific. Moreover, probiotics can release antioxidant enzymes and active compounds, such as SOD, CAT, NADH oxidase, TXN, and GSH, which are abundant in *Lactobacillus* and *Bifidobacterium*, indicating that probiotics have a substantial antioxidant activity (132, 133). Furthermore, probiotics modulate oxidative equilibrium by modulating antioxidant signaling pathways, with antioxidant effects exerted *via* the Nrf2, SIRT1, MAPK, and PKC pathways, demonstrating strain specificity (134). Finally, by strengthening the integrity of the intestinal barrier, initiating the immune response, and avoiding inflammation and OS, probiotics and their metabolites can prevent additional microbial metabolites and endotoxins from entering the circulation (135).

Probiotics can lower the incidence of BPD in premature children with a PMA of <32 weeks, demonstrating the potential of probiotics in the treatment of BPD (136). Probiotics can enhance the recovery of the gut-lung axis, anti-inflammation, and anti-infection by modulating the balance of gut microbiota and their remarkable antioxidative powers. However, probiotics can also have adverse effects on the human body. Thus, the safety of probiotics in the human body should be carefully examined before using them to treat infant with BPD, and the species, dose, and frequency of administration should be carefully chosen (137, 138). Formula is an excellent substitute for human milk when new mothers cannot breastfeed owing to physical limitations. Formula adds a range of nutrients to approximate human milk constituents while meeting the nutritional needs of neonates. In addition to specific protein components, fatty acids, carbohydrates, probiotics, and prebiotics are added to formula to control the microbiota of infants (139).

### HMOs

HMOs are the third most abundant solids in human milk, after lactose and fat. HMOs have potent anti-inflammatory, anti-infection, and prebiotic properties. They can also modulate the intestinal epithelial cell response and promote normal neonatal development through interactions with the gut flora. HMOs also play significant roles in adult brain development and cognitive function. The HMO content in mature human milk is approximately 12–15 g/L, and it gradually declines from colostrum to mature milk (140). Studies have also demonstrated that mothers of highly malnourished newborns have lower levels of validated acidic HMOs and fucosylated neutral HMOs than those of normal newborns (141). HMOs in human milk can bind to lactose to form an indigestible trisaccharide or tetrasaccharide; sialic acid can be added to create sialyllactoses (3′-SL and 6′-SL) and fucose can be added to form fucosyllactoses (2′-FL and 3-FL) (142).

The most prevalent oligosaccharide in human milk is 2′-FL. It comprises 30% of HMO. Tu et al. demonstrated that 2′-FL could react with whey protein and remove 2, 2-diphenyl-1-hydrazide radicals, resulting in high antioxidant activity. Further, 2′-FL-fortified formula improves intestinal protection, is well accepted by newborns, and has absorption and excretion rates comparable to those of human milk (143). Lacto-N-neotetraose (LNnT) is a neutral HMO that is not fucosylated. Both 2′-FL and LNnT are new prebiotic additives in formula that make the composition of formula similar to that of human milk. They demonstrate clinical benefits in terms of intestinal probiotic protection, immunological modulation, and brain development and are a safe addition to infant formula (122, 144). Another HMO found in human milk and fermented formula is 3′-galactosyl lactose (3′-GL), which inhibits NF-κB inflammatory signaling and preserves the intestinal barrier. As 3′-GL is a by-product of fermented formula, its safety has been established over time (145).

Most HMOs can reach the intestine, operate as metabolic substrates for the intestinal flora, interact with the intestinal flora, and play a role in modifying the immune system and eliminating pathogens in newborns with low milk oligosaccharide absorption rates. HMOs also act as prebiotics, encouraging the development of newborn intestinal *Bifidobacterium* and enhancing SCFA synthesis (146). HMOs can also produce butyrate when they react with intestinal bacteria (147). Given the critical role of HMOs in the development and antioxidant capacity of the intestinal flora, more forward-looking, thorough clinical research is needed to evaluate the association between HMOs and BPD.

### MFGM

MFGM is a fat droplet that is essential for lipid transport in human milk. It comprises three layers—phospholipid (PL), sphingomyelin (SM), and various protein–membrane structures. PL and SM are derived from alveolar epithelial cells. PL comprises the main glycerol chain, whereas SM is composed of the main ceramide chain. MFGM promotes proper brain development by improving neonatal immunological function, controlling the intestinal flora, and boosting neonatal immune function (148, 149). MFGM can interact with intestinal probiotics, increase the adhesion of probiotic bacteria in the gut, and increase residence time in the gut (150). The addition of MFGM to formula can help minimize variations in the intestinal flora between breastfeeding and formula feeding (151). MFGM supplementation can assist in maintaining the integrity of intestinal epithelial cells and reducing inflammatory responses in mice with lipopolysaccharide-induced inflammation, implying that MFGM has anti-inflammatory properties (152). An increasing number of trials involving the addition of MFGM to formula have been conducted in recent years. Clinical studies have indicated that MFGM in formula is safe and well tolerated, with few side effects, similar to those observed in breastfeeding babies (120, 123). There are currently no recommended trials on MFGM supplementation in infants with BPD.

### SCFAs

SCFAs, such as acetic acid, propionic acid, and butyric acid, are created by human gut flora metabolism and play a significant role in metabolism, the immune system, and anti-inflammation (153). The amount of SCFAs in human milk varies throughout the lactation period. Mature milk has the highest amount of SCFAs, which is four to seven times that in colostrum and transitional milk (105). SCFAs in human milk have been linked to newborn weight gain and obesity (154). SCFAs can enhance lung development and prevent lung disorders through the gut-lung axis. For example, butyrate, with a concentration of approximately 0.75 mM in human milk, protects the integrity of the intestinal barrier and significantly reduces mitochondrial damage in mouse models. OS is controlled by reducing hydrogen peroxide release and regulating the expression of related enzymes (155). Propionate plays a clear role in maintaining the pulmonary immune response; an increase in propionate in the intestinal flora has an anti-inflammatory effect (156). In a previous study, lung inflammation was improved, inflammatory factor expression was reduced, and intestinal microbiota was regulated in mice with BPD treated with acetate (157). In an Nrf2-dependent manner, sodium propionate can reduce lung inflammation and OS and facilitate alveolar simplification and aberrant angiogenesis generated by lipopolysaccharide in mice (158). These findings suggest that SCFAs are crucial for treating lung disorders. Pulmonary OS in newborns with BPD can encourage the production of inflammatory factors and trigger an inflammatory response, aggravating OS. SCFAs have been shown to reduce pulmonary inflammation, which is beneficial for treating BPD. Therefore, SCFAs are essential metabolites of the gut flora. Infants with BPD have a maladjusted gut flora, and human milk can deliver other SCFAs to neonates with BPD.

### Lactoferrin

Lactoferrin is a transferrin found in human and other mammalian milks. Lactoferrin is the most abundant protein in colostrum and possesses antibacterial, antioxidant, antiviral, and anticancer activities (159). Lactoferrin protects the body from OS in two ways—[1] it reduces the Fenton reaction and ROS production by binding to iron and preventing lipid peroxidation (106) and [2] it activates the NF-κB/MAPK pathway to relieve cellular inflammation, maintain cell barrier integrity, reduce OS, activate Nrf2 expression, upregulate GSH activity, and reduce ROS and MDA production (160). Lactoferrin is well tolerated in very young newborns, is straightforward to administer, and serves as a reference for lactoferrin supplementation in clinical practice (161). Lactoferrin supplementation for very young newborns has been shown to minimize the risk of neonatal necrotizing enterocolitis and late sepsis (162, 163). However, some studies have refuted this finding, proposing that enteral supplementation of lactoferrin does not reduce the incidence of infection or other diseases in premature infants and does not reduce the incidence of BPD. The mechanism of lactoferrin in the prevention of BPD is unclear (124). In a study by Dobryk et al., enteral lactoferrin supplementation at 100 mg/day did not affect BPD morbidity and death in preterm infants at PMA ≤32 weeks. However, it may help achieve faster completion of enteral feeding and a shorter hospital stay in the most premature newborns (164).

### Vitamins

#### Vitamin A

Vitamin A is a fat-soluble vitamin with biological activity and is a principal exogenous antioxidant. The lung is a critical target organ for vitamin A. Vitamin A can promote pulmonary vascular development, remove overabundant oxidative free radicals, and inhibit lipid peroxidation of the cell membrane, thereby reducing the effects of hyperoxia on lung development at various stages of alveolar development (165). Many studies have examined the critical role of vitamin A in the etiology of BPD. However, opinions on the prevention and treatment of BPD with early vitamin A supplementation are divided. In a clinical trial, early oral vitamin A (5,000 IU vitamin A/kg/day) minimized BPD morbidity and mortality in extremely low-birth-weight infants 28 days after delivery (126). In a study of very-low-birth-weight infants, Chabra et al. found that intramuscular injections of vitamin A can lower the incidence of BPD (166). High-quality reviews have demonstrated that early vitamin A supplementation for preterm newborns has good efficacy and safety, especially for extremely low-birth-weight infants. Vitamin A treatment can also reduce oxygen dependence for infants at a PMA of 36 weeks. However, as this supplementation effect has only been linked to a lower risk of BPD and not to lower risk of early death, more clinical research on amount and delivery of vitamin A supplementation is needed (167–170).

Although Abhijeet et al. demonstrated that enteral supplementation of water-soluble vitamin A improved plasma retinol levels in premature infants, they were unable to confirm a preventive or therapeutic effect on BPD, which might have been due to newborns' limited intestinal absorption capacity (171–173). The authors claimed that vitamin A supplementation might have a baseline—only vitamin A intake of 1,500 IU/kg/day was shown to reduce the incidence of BPD, regardless of the route of administration (enteral and parenteral) (174). In a phase III randomized controlled trial of 807 infants in 14 university hospitals across the United States, Matthew et al. found that vitamin A treatment reduced BPD morbidity and mortality in low-risk infants more than those in high-risk infants (175). Therefore, vitamin A treatment should not be restricted to more than those in high-risk neonates. Craig et al. discovered that nebulized vitamin A is more effective than injectable vitamin A in reducing hyperoxia-induced lung injury and improving BPD treatment (176). Human milk is also rich in vitamin A; therefore, it can exert a therapeutic effect through intestinal pathways.

#### Vitamin E

Vitamin E is a powerful antioxidant that eliminates free radicals from the human body. Human milk contains alpha- and gamma-tocopherol vitamin E. While infant vitamin E oral preparations are well tolerated throughout pregnancy, several investigations have found that vitamin E levels at birth are much lower in infants with BPD than in normal infants. Vitamin E insufficiency is inversely related to the period in which premature newborns require supplemental breathing, demonstrating an association between vitamin E deficiency and BPD severity (177). Most vitamin E clinical trials were halted in the 1990s, with several trials concluding that vitamin E had no evident clinical therapeutic effect on BPD. Due to the limitations of the research circumstances at the time and other considerations, such as other early diseases and understanding of BPD, the clinical application of vitamin E has not been extensively recognized (178–181).

However, compared to 30 years ago, qualitative changes in the understanding of BPD have occurred, namely, the revision from old BPD definition to the new BPD definition. With the improvement of premature infant nursing technology, birth gestational age has been raised from 30 weeks to around 26 weeks for children with BPD. At this time, it is in the tubular lung development or early capsule stage. The clinical characteristics of the new BPD were also altered from those of old BPD—“significant lung injury” was revised to “lung developmental arrest.” In the contemporary BPD context, it may be argued that research on vitamin E treatment for BPD has lost clinical importance. However, vitamin E supplementation has recently been shown to have a preventive effect on new BPD. BPD can be reduced by increasing the amount of vitamin E in the diet (182).

### Phytochemicals

Phytochemicals are a broad range of biochemical molecules produced by plants, but not by the human body. Lactating women can obtain phytochemicals through their diet, resulting in the production of breast milk phytochemicals. Human milk phytochemicals contain polyphenols and carotenoids, which have been shown to have strong antioxidant properties (183).

#### Polyphenols

Polyphenols are secondary metabolites with a polyphenolic structure found in plants. Flavonoids are an important subgroup of polyphenols, but there are no sources of polyphenols in the body. The amount of polyphenols in breast milk depends on the mother's food intake and absorption (184). Song et al. found epicatechin, epicatechin gallate, epicatechin gallate, naringin, kaempferol, hesperidin, and quercetin in the breast milk of full-term mothers (114).

Polyphenols, which are powerful antioxidants, have been shown to impact signal transmission and modulate intestinal enzyme activity through redox reactions. Polyphenol levels *in vivo* are inversely proportional to MDA, a lipid peroxidation product, implying that polyphenols can mitigate the effects of neonatal lipid peroxidation. Poniedziaek et al. discovered that nursing mothers who eat more vegetables had more polyphenols in their milk and higher antioxidant capacity (185). Currently, researchers are paying increasing attention to the antioxidant ability of polyphenols and flavonoids to avoid OS induced by ROS accumulation and diseases associated with OS. Polyphenol supplementation reduces chronic oxidative cell damage, DNA damage, inflammation, infection, and neurodegenerative disorders (186). Flavonoids have three main antioxidant effects— (1) metal chelation through different flavonoid structures under specific PH values, (2) reducing free radicals (superoxide, hydrogen peroxide, alkoxy) generated through hydrogen donation, where the newly generated free radicals react with other free radicals to form stable quinone structures, and (3) inhibiting oxidases (such as xanthine oxidase, microsomal oxygenase, and lipoxygenase) (187). Supplementing polyphenols in breast milk is a convenient and safe way to absorb them, but there is currently limited research on neonatal plasma polyphenol levels. And there is no evidence that polyphenols have positive effects for development of neonatal lungs, long-term clinical trials are indispensable to determine the benefits of polyphenols for newborns.

#### Carotenoids

Carotenoids are phytochemicals with antioxidant properties that can reduce the risk of cancer and cardiovascular disease. They are essential for immunological modulation and anti-aging. Beta-carotene can also be converted to the most common form of vitamin A in the body. Carotenoids and vitamin A levels in children with BPD are lower than expected (188). Carotenoid concentrations in breast milk change significantly over time, with α-carotene, β-carotene, alpha-cryptoxanthin, β-cryptoxanthin, zeaxanthin, lutein, and lycopene decreasing during early lactation (1-4 weeks) and then during the following nine weeks. As carotenoids are produced in breast milk during lactation and are consumed continuously after lactation, they do not accumulate in the mother and require exogenous supplementation (114). Given the high antioxidant capacity and ability of carotenoids to increase vitamin A levels in the body, it is reasonable to conclude that carotenoid supplementation is beneficial in treating BPD. Premature newborns can be administered carotenoids in human milk to boost their antioxidant capacity. However, clinical evidence of the advantages of carotenoids in newborn development and their impact on BPD is currently lacking.

### Melatonin

Melatonin is a hormone secreted by and retained in the pineal gland and subsequently processed in the liver. Melatonin secretion exhibits a distinct nocturnal circadian pattern. Melatonin is present in breast milk at higher concentrations at night and at lower concentrations during the day. Melatonin is a powerful endogenous antioxidant that can directly remove excess free radicals and promote the expression of other antioxidants such as SOD, CAT, and GPX (189). According to Li et al., melatonin can enhance the oxidative balance in mice with hyperoxia-induced lung injury by lowering MPO, nitrite/nitrate, and MDA levels; increasing GPX, CAT, and SOD activities; and reducing alveolar simplification and interstitial fibrosis. Given the similar lung development in mice and humans, it is plausible to assume that melatonin may protect against newborn hyperoxic lung injury (190). Selami et al. confirmed the therapeutic benefits of melatonin in rats with hyperoxia-induced lung damage. Melatonin treatment was found to increase the expression of lamellar protein and radiate alveolar count produced by AEC II, increase the levels of GPX and SOD, and decrease the levels of MDA, implying that melatonin can regulate the oxidative balance, protect pulmonary vascular endothelial cells, and promote alveolarization in hyperoxia-induced lung injury (191). For premature infants, melatonin treatment could reduce the mortality and hospital stay of BPD, which also need further studies to identify direct benefits of melatonin (125).

### Trace Elements

Trace elements are elements in the human body that contains <0.005–0.01% of body mass. Copper, selenium, iron, zinc, and other trace elements found in human milk form the foundation of the oxidation reaction of the body. Trace element supplementation can boost antioxidant function by catalyzing multiple redox processes by varying valence and activating endogenous antioxidants to eliminate ROS. Premature newborns acquire vital vitamins from human milk to fight excess reactive radicals. Trace elements are required by the body's antioxidant system; for example, selenium is necessary to maintain the activity of glutathione peroxidase, which is a powerful antioxidant that can remove lipid peroxidation. Selenium deficiency impairs the antioxidant function in the body. Supplementing premature newborns with selenium may improve their BPD prognosis, but it does not prevent BPD (192).

### Free Amino Acids

Free amino acids are amino acids that do not form peptides. Human milk contains a range of free amino acids, including arginine, a critical amino acid for premature newborns. Arginine is involved in the circulation of ornithine, which is crucial for immunological function and acid–base balance. The antioxidant properties of arginine are of interest. L-arginine has been shown to lower OS in the exercise state through NO production (193). The combined use of inhaled NO and vitamin A supplementation can minimize morbidity and mortality in premature infants who require early mechanical ventilation (194). *In vivo*, arginine is the raw material for GSH synthesis, and L-arginine supplementation can enhance GSH production and activate the Nrf2–ARE pathway, resulting in endogenous antioxidant responses (195).

Glutamine is a conditionally essential amino acid that can help build muscles, boost the immune system, and expand antioxidant capacity. Exogenous glutamine supplementation for a short period can increase serum CAT and SOD activities and has an antioxidant effect. Glutamine has been shown in some studies to protect mice from hyperoxia-induced lung injury. Arginine–glutamine dipeptide is a stable source of water-soluble glutamine and protects newborn mice from hyperoxic lung injury (196). But there are not direct researches to study the benefits of free amino acids in BPD.

### Antioxidant Enzymes and GSH

Antioxidant enzymes are important substances for human body to against OS. Human milk contains antioxidant enzymes such as SOD, CAT, and GPX, which are crucial for preventing lung damage in neonates. SOD catalyzes the conversion of superoxide anion free radicals to hydrogen peroxide. In contrast, CAT and GPX are responsible for hydrogen peroxide breakdown and prevention of oxidative free radical accumulation in the body. Excess ROS cannot be removed in premature newborns due to abnormalities in the endogenous antioxidant enzyme system, leading to ROS accumulation and, ultimately, OS. Breastfeeding can improve the antioxidant capacity of premature infants. Colostrum has a higher antioxidant capacity than transitional milk or mature milk, and it can aid in the early transition from intrauterine hypoxia to extrauterine hyperoxia and improve the antioxidant system (189).

Human milk also contains GSH, a crucial non-enzymatic antioxidant. GSH participates in the circulation of vitamins C and E, boosting antioxidant capacity and engaging in several redox processes as a reducing agent. GSH levels in the alveolar lavage fluid of children with BPD have been shown to be considerably lower than those in children without BPD. A survey of GPX-deficient neonatal mice revealed that increased GSH-dependent redox reactions could lower oxidative lung damage and the prevalence of BPD (71).

## Discussion

BPD is a chronic respiratory condition with long-term detrimental implications for newborn health, such as reduced quality of life and poor clinical outcomes. Awareness of BPD has grown in recent years due to ongoing research on its etiology and pathogenesis. OS due to hyperoxia exposure is a significant risk factor for BPD. There are additional preventative and therapeutic targets for BPD. Antioxidant treatment has become an essential strategy for the adjuvant treatment of preterm newborns with BPD despite the lack of standardized clinical signs. OS has a range of effects on normal lung development. When premature infants are born, their lungs are still in the growth stage, and hyperoxia causes poor lung development, increases death of alveolar epithelial cells, and triggers pulmonary vascular remodeling. Although OS plays a role in the development of BPD, there are several limitations to clinical oxidation treatment—treatment is limited to a single type of antioxidant in clinical practice, antioxidant treatment is used only as a supplement to conventional treatment of BPD, and a poor understanding of antioxidant treatment level. However, studies on the degree of antioxidant therapy in preterm newborns using exogenous antioxidants are scarce. Determining how to target the OS site to administer exogenous antioxidant molecules is challenging, which directly impacts the stability and efficacy of antioxidant therapies. Therefore, clinical antioxidant therapy often does not have the desired effect. Antioxidant treatment is undeniably effective in combating oxidative imbalances. Inhibition of OS *via* the Nrf2, SIRT1, and TXN signaling pathways has shown promising results in animal models, demonstrating the potential of antioxidant therapy in preventing and controlling BPD. More clinical trials are needed to confirm the association between OS and BPD and further investigate the benefits of antioxidant therapy for BPD.

Human milk is the most natural and safe food for infants. The current review reveals that breastfeeding may help minimize the risk of developing BPD, although the role of human milk in preventing and treating BPD is unknown. Breastfeeding has many beneficial effects on BPD. Human milk delivers a high level of nutrients that aid in the normal development of premature newborns after birth, improve their nutritional status, and encourage the continuous development of their lungs. Furthermore, breastfeeding reduces postpartum inflammation, improves immunity, reduces newborn infections, and lowers the incidence of BPD. Human milk also contains several antioxidants that are thought to minimize OS in the BPD process. In this review, we examined several active compounds in human milk, considering them from different perspectives to collate how they may ameliorate BPD OS in lung development as follows: 1. direct secretion of antioxidant substances clears free radicals and prevents their accumulation, 2. metal chelation, 3. enhancement of the antioxidant signaling pathway, 4. interaction with the intestinal flora, 5. inhibition of lipid peroxidation, and 6. anti-inflammatory and anti-infection properties.

Human milk has a high antioxidant capacity, which supports the benefits of breastfeeding for preterm neonates. However, there are several limitations to this view. Firstly, we identify only the antioxidants that play an antioxidant role in BPD. It is unclear whether they act as effective antioxidants in breast milk and whether there is a synergistic effect between these substances. Secondly, most studies cited in this view associated with the antioxidant effect of BPD do not come from breast milk OS. The research about breast milk antioxidants and their effects on BPD are still limited based on ethical factors. thirdly, there are limited data on human milk from mothers of premature infants; therefore, differences in antioxidant capacity compared to that of human milk from mothers of full-term infants has not been clearly shown. Owing to ethical considerations, few randomized controlled trials to prevent premature BPD have been conducted. Hence, observational studies have been given precedence.

While the effects of breast milk on BPD have been addressed, there are much research in this territory that require future studies. In this view, we suggest researchers take these issues into account: 1. Should the combination of probiotics be added to the formula? 2. Does the antioxidants in breast milk have synergistic effects when breastfeeding? 3. What are the positive effects of MFGM on antioxidation? 4. What roles does the gut-lung axis play when SCFAs affect as antioxidative substances? 5. Do the Vitamin E treatments have effects accompanied by the evolution of BPD? 6. How does the addition of polyphenols and carotenoids to the formula impact the development of neonatal lungs? 7. Does melatonin have effects on BPD in preterm infants? These controversies require further clinical studies although the limitations are still hard. And we also noticed that stem cells in breast milk present a prominent potential for BPD treatment, it is worth studying their antioxidative effects on BPD.

Individual variables related to human milk, such as neonatal birth gestational age, whether the mother is breastfeeding, and whether exclusive breastfeeding is practiced, can have a significant impact on the composition of human milk. Human milk from mothers of premature newborns differs substantially from human milk from mothers of full-term newborns. Chrustek et al. found that compared to human milk from mothers of full-term newborns, that from mothers of premature infants may contain more antioxidants. Premature newborns with a higher total antioxidant status are protected from free radicals, preventing ROS accumulation and OS (197). Antioxidants in human milk have been modified to the PMA to provide improved protection for premature newborns. Some active compounds in breast milk can increase the antioxidant capacity of premature infants, which is conducive to the rapid establishment of oxidative balance in premature infants with insufficient antioxidant capacity.

## Conclusion

Human milk contains many bioactive compounds that supply essential nutrients to newborns and act as antioxidants to protect premature babies from OS. Existing research suggests that breastfeeding can ameliorate the effects of BPD. However, more high-quality tests and studies are needed to provide evidence for the role of breastfeeding in the prevention and control of BPD. Breastfeeding is recommended for full-term and preterm infants, although their antioxidant capacities differ. High-dose breastfeeding has the potential to be an effective and inexpensive treatment option for BPD.

## Author Contributions

XY is responsible for the collection of data and writing of the original manuscript. SJ is responsible for the organization of the original manuscript. XD and ZL are responsible for editing. AC and RY are responsible for the concept development, revision, review of the manuscript, and funding acquisition. All authors contributed to the article and approved the submitted version.

## Funding

This research was supported by National Natural Science Foundation of China (No. 82101812), Jiangsu Commission of Health and Family Planning (No. Z2020042), Wuxi Medical Innovation Team (No. CXTD2021013), and Wuxi Young and Middle-aged Medical Talents Project (Nos. BJ2020075 and BJ2020079).

## Conflict of Interest

The authors declare that the research was conducted in the absence of any commercial or financial relationships that could be construed as a potential conflict of interest.

## Publisher's Note

All claims expressed in this article are solely those of the authors and do not necessarily represent those of their affiliated organizations, or those of the publisher, the editors and the reviewers. Any product that may be evaluated in this article, or claim that may be made by its manufacturer, is not guaranteed or endorsed by the publisher.

## Abbreviations

BPD: Bronchopulmonary dysplasia
OS: Oxidative stress
NICHD: The National Institute of Child Health and Human Development
ES: Endoplasmic reticulum stress
UPR: Unfolded protein response
VEGF: Vascular endothelial growth factor
MSC: Mesenchymal stem cells
Nrf2: Nuclear erythroid-E2-related factor 2
ARE: Antioxidant reaction elements
HO-1: Heme oxygenase-1
SIRT1: Silent mating-type information regulation 2 homolog 1
NAD: Nicotinamide adenosine dinucleotide
SENP1: Small ubiquitin-like modifier (SUMO)-specific protease 1
PBMC: Peripheral blood mononuclear cells
PPARγ: Peroxy-proliferator-activated receptor γ
AT2: Primary alveolar type 2 cells
TXN: Thioredoxin
TXNRD: Thioredoxin reductase
ATG: Aurothioglucose
NF-κB: Nuclear factor κB
Tet: Tetralin
ISAPP: The International Scientific Association for Probiotics and Prebiotics
2′-FL: 2′-fucosyllactose
LNnT: Lacto-N-neotetraose
3′-GL: 3′-galactosyl lactose
HMO: Human milk oligosaccharides
DPPH, 2: 2-diphenyl-1-hydrazide
SCFA: Short chain fatty acids
SOD: Superoxide dismutase
GSH-Px: Glutathione peroxidase
NQO1: NAD (P)H:quinone oxidoreductase.
